# Rescue therapy with inhaled nitric oxide and almitrine in COVID-19 patients with severe acute respiratory distress syndrome

**DOI:** 10.1186/s13613-020-00769-2

**Published:** 2020-11-04

**Authors:** François Bagate, Samuel Tuffet, Paul Masi, François Perier, Keyvan Razazi, Nicolas de Prost, Guillaume Carteaux, Didier Payen, Armand Mekontso Dessap

**Affiliations:** 1 AP-HP, Hôpitaux Universitaires Henri-Mondor, Service de Médecine Intensive Réanimation, 51, avenue du Maréchal de Lattre de Tassigny, 94010 Créteil, France; 2 Univ Paris Est Créteil, CARMAS, 94010 Créteil, France; 3 UFR de Médecine Villemin, Université Paris 7 Paris Cité Sorbonne, Paris, France; 4 Univ Paris Est Créteil, INSERM, IMRB, 94 010 Créteil, France

**Keywords:** iNO, Almitrine, COVID-19, ARDS

## Abstract

**Background:**

In COVID-19 patients with severe acute respiratory distress syndrome (ARDS), the relatively preserved respiratory system compliance despite severe hypoxemia, with specific pulmonary vascular dysfunction, suggests a possible hemodynamic mechanism for VA/Q mismatch, as hypoxic vasoconstriction alteration. This study aimed to evaluate the capacity of inhaled nitric oxide (iNO)–almitrine combination to restore oxygenation in severe COVID-19 ARDS (C-ARDS) patients.

**Methods:**

We conducted a monocentric preliminary pilot study in intubated patients with severe C-ARDS. Respiratory mechanics was assessed after a prone session. Then, patients received iNO (10 ppm) alone and in association with almitrine (10 μg/kg/min) during 30 min in each step. Echocardiographic and blood gases measurements were performed at baseline, during iNO alone, and iNO–almitrine combination. The primary endpoint was the variation of oxygenation (PaO_2_/FiO_2_ ratio).

**Results:**

Ten severe C-ARDS patients were assessed (7 males and 3 females), with a median age of 60 [52–72] years. Combination of iNO and almitrine outperformed iNO alone for oxygenation improvement. The median of PaO_2_/FiO_2_ ratio varied from 102 [89–134] mmHg at baseline, to 124 [108–146] mmHg after iNO (*p* = 0.13) and 180 [132–206] mmHg after iNO and almitrine (*p* < 0.01). We found no correlation between the increase in oxygenation caused by iNO–almitrine combination and that caused by proning.

**Conclusion:**

In this pilot study of severe C-ARDS patients, iNO–almitrine combination was associated with rapid and significant improvement of oxygenation. These findings highlight the role of pulmonary vascular function in COVID-19 pathophysiology.

## Introduction

Severe acute respiratory syndrome coronavirus 2 (SARS-CoV-2) which is responsible for the coronavirus disease 2019 (COVID-19) pandemic is causing a massive influx of patients presenting with severe acute respiratory distress syndrome (ARDS) to intensive care units (ICUs) worldwide [1]. For the most severe cases, refractory ARDS may lead to a discussion regarding the use of extracorporeal membrane oxygenation (ECMO), an expensive and invasive life support resource, available in limited numbers in expert centers [2, 3]. Since the possibilities cannot fit with the large-scale outbreaks, alternative solutions should be proposed [4].

Some authors have hypothesized that potential relatively preserved respiratory system compliance (Crs) despite severe hypoxemia in COVID-19 patients suggests a possible hemodynamic mechanism for ventilation/perfusion (VA/Q) mismatch as hypoxic vasoconstriction alteration [5]. The SARS-COV-2 uses angiotensin converting enzyme 2 (ACE2) receptor expressed by pneumocytes in the epithelial alveolar lining to infect the host, thereby causing lung injury, but the ACE2 receptor is also widely expressed on endothelial cells, including the heart, kidney, intestine and lung. The presence of viral elements within endothelial cells with an accumulation of inflammatory cells, suggest that SARS-CoV-2 infection may induce endotheliitis altering vascular reactivity [6] including the hypoxic vasoconstriction or other vasomotion control.

The combination of inhaled nitric oxide (iNO), a selective pulmonary vasodilator, and almitrine, a specific pulmonary vasoconstrictor, was proposed several decades ago as to improve VA/Q mismatch. It was spectacular in many ARDS patients with maintained vasodilation in ventilated zones receiving iNO and reduced perfusion in poorly or non-ventilated zones after almitrine treatment [7–9]. In the particular context of COVID-19, we hypothesized that iNO–almitrine combination could improve arterial oxygenation in severe COVID-19 ARDS (C-ARDS) by a redistribution of the pulmonary blood flow towards ventilated areas.

## Methods

### Patient selection

Intubated patients with laboratory-confirmed COVID-19, who met the criteria for ARDS (Berlin definition) [10] with persistent severe hypoxemia (PaO_2_/FiO_2_ < 150 mmHg), were prospectively included at the medical ICU of Henri Mondor University Hospital (Creteil, France). SARS-CoV-2 infection was confirmed by real-time reverse transcriptase-polymerase chain reaction (RT-PCR) assay of nasal swabs or lower respiratory tract samples (bronchoalveolar lavage or endotracheal aspirate). Age lower than 18 years, acute cor pulmonale defined as septal dyskinesia with dilated right ventricle (end-diastolic right ventricle/left ventricle area ratio > 0.6), pulmonary embolism, hyperlactatemia (> 2 mmol/L), hepatic insufficiency, and ECMO support were exclusion criteria. Respiratory settings and ARDS management were in accordance with French guidelines [11]. The study was approved by the ethics committee of the French Intensive Care Society as a component of standard care, and patient consent was waived as per French law. Families were given information about the study.

### Protocol

Enrolled patients were sedated and received neuromuscular blockers to maintain a volume-control mechanical ventilation adapted to keep the tidal volume around 6 mL/kg of predicted body weight (PBW) and the PaCO_2_ below 50 mmHg. After hemodynamic and ventilatory optimization, prone positioning was tested because of persisting severe hypoxemia (PaO_2_/FiO_2_ < 150 mmHg). After a proning session lasting 16 to 18 h, the patients were put back to supine position and the iNO (10 ppm) alone followed by iNO associated with 10 mcg/kg/min of almitrine (Vectarion®, Servier, Suresnes, France) were tested. The FiO_2_ was settled at 1 to limit heterogeneity within patients and to look at the effect of the drugs on true Qs/Qt, eliminating mostly the low VA/Q zones. The effect on arterial oxygenation was evaluated at least after 30 min in each condition: supine baseline, iNO, and iNO plus almitrine. Because of the potential negative impact of right ventricle afterload increase during almitrine, the right ventricular function was assessed by echocardiography along with arterial blood gases at baseline, during iNO alone, and with iNO–almitrine combination. Patients who had a PaO_2_/FiO_2_ ratio that increased by at least 20% or by 20 mmHg as compared to the baseline situation were considered “responders” [12].

### Respiratory mechanics

The assessment of respiratory mechanics included the following measurements. Plateau pressure and total PEEP were assessed during an end-inspiratory (0.3 s) and end-expiratory (1–2 s) occlusion maneuver, respectively. The driving pressure and the Crs were computed as the difference between plateau pressure and total PEEP and tidal volume divided by the difference between plateau pressure and total PEEP, respectively. The potential airway closure phenomenon was detected by measuring the airway opening pressure during a low flow (≤ 6 L/min) insufflation and potential for lung recruitment was assessed by the mean of the recruitment-to-inflation ratio (R/I ratio) computation, as previously described by Chen et al. [13]. A R/I ratio < 0.5 was used to characterize a poorly recruitable patient.

### Echocardiography

Trained operators (competence in advanced critical care echocardiography) performed transthoracic echocardiography in the supine position at baseline, and during iNO and almitrine administration. They focused on global function (velocity–time integral of left ventricular outflow tract, cardiac index), and the right ventricle function as previously proposed [14]. Because of severe hypoxia, all patients had a detection of potential shunting across patent foramen ovale in four-chamber view after injection of sterile-modified fluid gelatine solution (Plasmion, Fresenius-Kabi, Sevres, France) aerated with room air to generate microbubbles as previously proposed [15].

### Other variables collected

The following data were collected at inclusion: age, gender, body mass index, past medical history, standard treatments, Charlson comorbidity index, Sequential Organ Failure Assessment (SOFA) score [16], Simplified Acute Physiologic Score (SAPS) II [17], and the need for vasopressors. In addition, the need for ECMO support, limitation of life-sustaining therapies and ICU mortality were collected during hospitalization.

### Statistical analysis

Statistical analyses were performed with the JMP software (version 9; SAS Institute Inc, Cary, NC) and GraphPad Prism software (version 5; GraphPad Software Inc., La Jolla, CA, USA). The primary endpoint of this study was the variation of oxygenation (PaO_2_/FiO_2_). Data were presented as median with interquartile range or number with percentage. Multiple paired values were compared using Friedman test followed by paired Wilcoxon test with Benjamini–Hochberg correction. Spearman’s test was used to assess correlation. For all tests, a two-way *p*-value < 0.05 was considered statistically significant.

## Results

### Patients characteristics

As a pilot study, ten severe C-ARDS patients were assessed (seven males and three females), with a median age of 60 [52–72] years. Median time since endotracheal intubation was 7 [5–15] days, allowing to mix potential different hypoxic mechanisms. Clinical characteristics, comorbidities, standard treatments and organ failures at inclusion are presented in Table 1.

**Table 1:** Clinical characteristics of ten patients with Coronavirus 19 severe acute respiratory distress syndrome

Variables	
Age (years)	60 (52–72)
Female gender	3 (30%)
Body mass index (kg/m^2^)	28.2 (22.0–37.4)
Comorbidities	
Dyslipidemia	2 (20%)
Current smoker	2 (20%)
Arterial hypertension	5 (50%)
Diabetes mellitus	5 (50%)
Atrial fibrillation	0
Heart failure	0
Previous stroke	0
COPD	1 (10%)
Cirrhosis	0
Dialysis	1 (10%)
Previous stroke	0
Standard treatments	
Anticoagulant	0
Antiplatelet therapy	0
ACE inhibitors or ARB	5 (50%)
Organ failure at admission	
Charlson comorbidity index	2 (0–2)
SAPS-II	36 (32–46)
SOFA score	6 (3–7)
PaO_2_/FiO_2_	112 (92–144)
Neuromuscular-blocking agent use	7 (70%)
Shock	1 (10%)

Values are expressed as median (interquartile range)

*C-ARDS* COVID-19 ARDS patients, *ARDS*: acute respiratory distress syndrome, *COPD* chronic obstructive pulmonary disease, *ACE* angiotensin-converting enzyme, *ARB* angiotensin receptors blockers, *SAPS II* Simplified Acute Physiology Score II, *SOFA score *Sequential Organ Failure Assessment, *PaO*_*2*_ arterial oxygen tension, *FiO*_*2*_ fraction of inspired oxygen

## Prone session and respiratory mechanics

As shown in Additional file 1: Table S1, the gas exchange response of the last prone position the day before the protocol was favorable (increase in PaO_2_/FiO_2_ of at least 20% or 20 mmHg) in most (8/10, 80%) patients; overall, the PaO_2_/FiO_2_ ratio increased from 77 [62–114] mmHg (supine) to 137 [97–167] (prone), *p* < 0.01 (Additional file 1: Table S1). Respiratory mechanics in supine position after proning are reported in Additional file 2: Table S2. The median values of Crs and driving pressure were 28 [21–38] mL/cmH_2_O, and 15 [12–16] mmHg, respectively. R/I ratio was < 0.5 in 6/10 (60%) patients, indicating limited recruitability in a majority of patients.

### Effect of iNO and almitrine

**Fig. 1:**
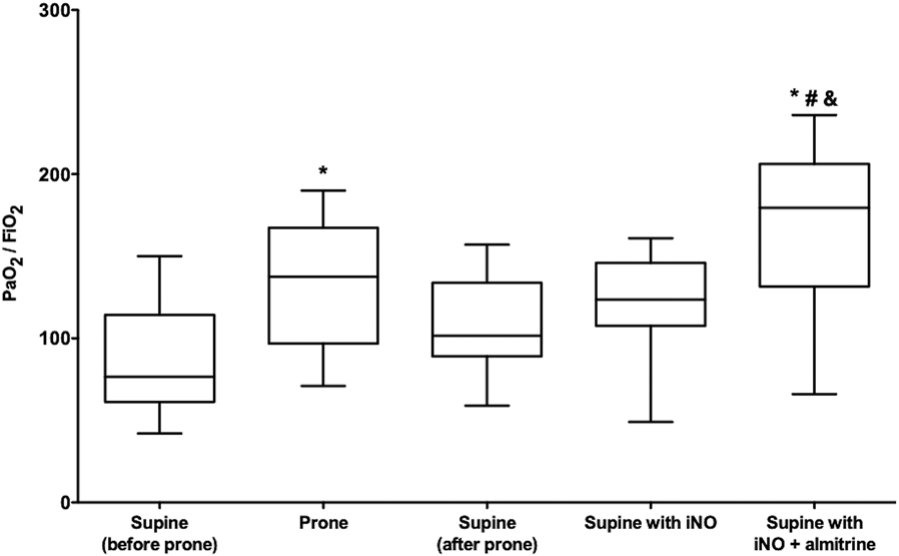
Box and whiskers plots of the change in the ratio of oxygen partial pressure (PaO_2_) to fraction of inspired oxygen (FiO_2_) in arterial blood in prone position and supine position, before and after administering inhaled nitric oxide (iNO) and almitrine in patients with severe acute respiratory distress syndrome secondary to coronavirus disease 2019. *, ^#^, and ^&^ denote a *p* value < 0.05 for paired Wilcoxon (with Benjamini–Hochberg correction) following Friedman test, as compared to supine (before prone), supine (after prone), and supine with iNO, respectively

In supine position, patients were still severely hypoxic with median PaO_2_ of 102 [89–134] mmHg at FiO_2_ of 1 (Table 2). On supine position, only the addition of almitrine to iNO increased significantly PaO_2_ from baseline (Fig. 1), with no significant changes in pulmonary blood flow and other hemodynamic and echocardiographic variables (Table 2). The median of PaO_2_/FiO_2_ ratio increased from 102 [89–134] mmHg at baseline, to 124 [108–146] mmHg after iNO (*p* = 0.13) and 180 [132–206] mmHg after iNO and almitrine (*p* < 0.01) (Table 2). PaO_2_ increased by more than 50% in seven of ten patients with iNO–almitrine combination (Additional file 3: Figure S1). One non-responder had an intra-cardiac shunt related to patent foramen ovale. The response to iNO + almitrine did not correlate with the benefit on PaO_2_ induced by prone positioning (ρ = −0.09, *p* = 0.80). Similarly, the baseline respiratory mechanics were not associated with the iNO–almitrine response (Additional file 4: Table S3).

**Table 2:** Clinical data, arterial blood gases, and echocardiographic findings with the administration of inhaled nitric oxide (iNO) and almitrine in patients with severe acute respiratory distress syndrome secondary to coronavirus disease 2019

Variables	Baseline	iNO	iNO + almitrine
Clinical parameters			
Systolic arterial pressure (mmHg)	141 (122–148)	131 (115–146)	131 (117–145)
Diastolic arterial pressure (mmHg)	58 (56–73)	60 (51–73)	57 (47–74)
Mean arterial pressure (mmHg)	82 (66–94)	80 (70–95)	78 (73–96)
Heart rate (rpm)	102 (86–111)	95 (86–117)	93 (87–116)
Pulse oxygen saturation (%)	98 (95–99)	98 (97–99)	100 (98–100)^a,b^
Arterial blood gas			
FiO_2_	1	1	1
pH	7.30 (7.29–7.36)	7.32 (7.30–7.37)^a^	7.32 (7.30–7.39)^a^
PaO_2_ (mmHg)	102 (89–134)	124 (108–146)	180 (132–206)^a,b^
PaCO_2_ (mmHg)	48 (40–55)	45 (40–60)	46 (39–56)
Bicarbonates (mmol/L)	26 (23–32)	26 (22–32)	28 (23–31)
SaO_2_ (%)	97 (94–97)	98 (96–98)	99 (98–99)^a^
Lactate (mmol/L)	1.1 (0.9–1.5)	1.0 (0.9–1.6)	1.1 (0.9–1.8)
Echocardiographic parameters			
VTI LVOT (cm)	17 (15–22)	16 (15–21)	20 (17–25)
Cardiac index (L/min/m^2^)	3.2 (2.2–4.1)	2.8 (2.2–3.6)	2.9 (2.7–4.2)
TR velocity peak (m/s)	2.8 (2.1–3.1)	2.2 (2.0–2.9)	2.7 (2.1–3.0)
LV eccentricity index	0.95 (0.9–1.0)	0.97 (0.92–1.0)	0.97 (0.84–1.0)
RV/LV surface ratio	0.5 (0.41–0.54)	0.44 (0.37–0.57)	0.49 (0.45–0.63)

Values are expressed as median (interquartile range)

*iNO* inhaled nitric oxide, *FiO*_*2*_ fraction of inspired oxygen, *PaO*_*2*_ arterial oxygen tension, *PaCO*_*2*_ arterial carbon dioxide tension, *SaO*_*2*_ arterial oxygen saturation, *VTI LVOT* velocity–time integral of left ventricular outflow tract, *TR* tricuspid regurgitation, *LV* left ventricle, *RV* right ventricle

^a^Denotes a p value < 0.05 as compared to baseline, for paired Wilcoxon (with Benjamini–Hochberg correction) following Friedman test

^b^Denotes a p value < 0.05 as compared to iNO, for paired Wilcoxon (with Benjamini–Hochberg correction) following Friedman test. Baseline denotes supine position, a median of 4 [2–6] hours after end of last proning session

### Outcomes

Although the study was not designed to evaluate the impact on outcome, it is important to report that six out of ten patients had a refractory hypoxemia (PaO_2_/FiO_2_ < 80 mmHg), which could not be treated by almitrine due to the shortage of drug reserve. One patient benefited from ECMO support with a favorable final outcome, the five remaining could not be treated by ECMO and died during ICU stay.

## Discussion

The main findings of this pilot study were as follows: i) only the combination of iNO and almitrine improved the arterial oxygenation in severe C-ARDS patients; ii) the response to iNO–almitrine was not associated to the prone positioning effect and to the baseline respiratory mechanics variables.

In vitro studies suggested a direct antiviral effect of iNO on the SARS-CoV replication cycle [18, 19]. During the first SARS-CoV outbreak in 2004, a pilot study reported the efficacy of iNO in a limited series of severe patients, with reversal of pulmonary hypertension, improved hypoxemia and shortened duration of mechanical ventilation [20]. Some authors suggested that iNO could be used as a rescue therapy during the current pandemics [21, 22], inasmuch as COVID-19 is characterized by major pulmonary vascular dysfunction with endothelialitis, and thrombosis [23, 24]. In our case series, iNO alone had a negligible effect on oxygenation. In addition, in the absence of RV dysfunction, iNO did not change the RV 2D echocardiographic measures. These results confirm the adequate exclusion of patients with pulmonary hypertension and/or RV dysfunction, to safely use almitrine. Ongoing randomized controlled trial testing iNO will probably shed light on its usefulness in a broader population of patients with C-ARDS [25].

Some authors have hypothesized that in some patients with C-ARDS (especially those with low elastance–“L type”), hypoxemia was not completely explained by pulmonary shunt resulting from diffuse alveolar damage [26]. The respiratory mechanics of our selected patients did not fully match with the proposed “L type”, described by Gattinoni et al. [26], but was in accordance with a recent larger cohort of critically ill adults with COVID-19 [27]. The frequency of vascular and perfusion abnormalities [28] and pulmonary embolism incidence seems higher in COVID-19 pneumonia as compared to classical ARDS [29]. There is also a specific pulmonary procoagulant pattern [30], causing alveolar capillary microthrombi, as revealed by post-mortem studies [31, 32]. More interestingly, Ackermann et al. reported [31] the presence of intussusceptive angiogenesis. These anomalies may alter hypoxic pulmonary vasoconstriction, a possible mechanism for VA/Q mismatch and hypoxemia during C-ARDS. Addition of almitrine to iNO in patients with C-ARDS has the potential for restoring vascular homeostasis, in particular hypoxic pulmonary vasoconstriction [28].

The first reported study on almitrine in severe hypoxia in COVID-19 patients [33] showed a highly significant increase in P/F ratio with almitrine, independently from the dose used (4 or 12 mcg/kg/min). Because the level of PvO_2_ entering the pulmonary circulation is a major controller of hypoxic pulmonary vasoconstriction [34], they measured the SvO_2_, that increased significantly. Recently, Barthélémy et al. [35] described the effect of almitrine in 19 critically ill COVID-19 patients. In this study, almitrine (2 μg/kg/min) globally increased oxygenation within 6 h of infusion start. However, the studied population was heterogeneous, and the effect of prone position was not reported. Another study reported the effect of iNO (10 to 20 ppm in 10 patients), almitrine (0.5 mg/kg over 30 min in 13 patients), or both (7 patients). Surprisingly, the authors failed to observe any oxygenation improvement, with all patients investigated in prone position [36]. Taken together, previous reports and our study suggest a beneficial effect mainly during almitrine infusion in C-ARDS in the supine position.

In our study, since pulmonary blood did not change, it is reasonable to consider that the drugs combination creates pulmonary resistance gradient favoring the perfusion of ventilated areas reducing the VA/Q mismatch [37]. These data are consistent with previous larger studies in non-COVID ARDS [7, 8, 38]. Moreover, a recent preliminary study in non-COVID ARDS patients with veno-venous ECMO support, might renew the interest for almitrine [39]. The role of iNO and almitrine in the therapeutic arsenal of ARDS is not yet completely clear, but it is reasonable to consider iNO and almitrine as potential rescue therapies that might be applied in case of persisting severe hypoxemia despite prone positioning and before considering ECMO [11].

Our study suffers from several limitations. First, it is a pilot study on a small cohort, with no control group of ARDS not resulting from COVID-19, making the results only exploratory. However, our C-ARDS patients were homogeneous in terms of severity and selection. Second, because of limited drug availability, we did not evaluate the prolonged effect of this therapeutic combination. Thus, full interpretation on efficacy and tolerance is not possible. We did not observe adverse events on this short duration of administration. At least for a short duration, almitrine did not cause hyperlactatemia, hemodynamic instability (by favoring acute cor pulmonale), or hepatic disturbances [40]. Third, we could not standardize the timing of evaluation referring to prone position. A potential impact of additive effects of prone position and iNO-almitrine on arterial oxygenation cannot be ruled out [36]. Fourth, ventilation in FiO_2_ 1 may theoretically increase the alveolar partial pressure in oxygen and inhibit or at least decrease hypoxic pulmonary vasoconstriction in non- or hypo-ventilated areas. However, an FiO_2_ of 1 was used for the following reasons: i) the level of hypoxia for almost all patients necessitated very high FiO_2_ close to 1; ii) the FiO_2_ of 1 allows measuring hypoxia mainly related to true Qs/Qt and not low VA/Q zones. It is then more rigorous to compare the results of modification of true shunt instead of global venous admixture containing also low VA/Q; iii) the gas equation used to calculate the P/F ratio may introduce large bias as previously shown.

## Conclusion

In this small series of severe C-ARDS patients, the iNO–almitrine combination was associated with rapid and significant improvement of oxygenation, which was not observed with iNO alone. These findings highlight the role of pulmonary vascular vasoreactivity in COVID-19, which could partially be corrected by almitrine. This may help to avoid the ECMO or delay the time at which ECMO can be initiated. This aspect could only be evaluated in a randomized clinical trial in presence or not of almitrine. More work is warranted to test whether the prolonged use of these medicines could alter the long-term outcome of such patients.

## Supplementary information

**Additional file 1: Table S1.** Blood gas before and after the last proning session in ten patients with severe acute respiratory distress syndrome secondary to coronavirus disease 2019.

**Additional file 2: Figure S1.** Individual values of the ratio of oxygen partial pressure to inspired oxygen fraction in arterial blood in patients with severe acute respiratory distress syndrome secondary to coronavirus disease 2019, according to position (prone or supine) and administration of inhaled nitric oxide with or without almitrine. ^*^,^#^ and ^&^ denote a p value <0.05 for paired Wilcoxon (with Benjamini-Hochberg correction) following Friedman test, as compared to Supine (before prone), Supine (after prone), and Supine with iNO, respectively. Red lines: “almitrine non-responders”; blue lines: “almitrine responders”; solid lines: “prone responders”; dashed lines: “prone non-responders”.

**Additional file 3: Table S2.** Respiratory mechanics in supine position in ten patients with severe acute respiratory distress syndrome secondary to coronavirus disease 2019.

**Additional file 4: Table S3.** Correlations between respiratory mechanics and oxygenation response to the combination of inhaled nitric oxide and almitrine in ten patients with severe acute respiratory distress syndrome secondary to coronavirus disease 2019.

## Data Availability

All data generated and analyzed during the study are included in the published article and can be shared upon request. All authors helped to revise the draft of the manuscript. All authors read and approved the final manuscript.

## Abbreviations

Crs: Respiratory system compliance
ARDS: Acute respiratory distress syndrome
iNO: Nitric oxide
C-ARDS: COVID-19 ARDS
SARS-CoV-2: Severe acute respiratory syndrome coronavirus 2
COVID-19: Coronavirus disease 2019
ACE2: Angiotensin converting enzyme 2
ICU: Intensive care units
ECMO: Extracorporeal membrane oxygenation
Crs: Respiratory system compliance
VA/Q: Ventilation/perfusion
PBW: Predicted body weight
PaO_2_/FiO_2_: Arterial oxygen tension to fraction of inspired oxygen ratio
RT-PCR: Real-time reverse transcriptase-polymerase chain reaction
PEEP: Positive end-expiratory pressure
SOFA: Sequential Organ Failure Assessment
SPAS II: Simplified Acute Physiologic Score II
LV: Left ventricle
RV: Right ventricle
PaCO_2_: Partial pressure of arterial carbon dioxide
SaO_2_: Arterial oxygen saturation
VR: Ventilatory ratio
VCO_2_: Carbon dioxide production

## References

[1] Zhu N, Zhang D, Wang W, Li X, Yang B, Song J (2020). A novel coronavirus from patients with Pneumonia in China, 2019. N Engl J Med.

[2] DellaVolpe J, Barbaro RP, Cannon JW, Fan E, Greene WR, Gunnerson KJ (2020). Joint society of critical care medicine-extracorporeal life support organization task force position paper on the role of the intensivist in the initiation and management of extracorporeal membrane oxygenation. Crit Care Med.

[3] Ramanathan K, Antognini D, Combes A, Paden M, Zakhary B, Ogino M, et al. Planning and provision of ECMO services for severe ARDS during the COVID-19 pandemic and other outbreaks of emerging infectious diseases. Lancet Respir Med. 2020; 10.1016/S2213-2600(20)30121-1 PMC7102637 32203711

[4] MacLaren G, Fisher D, Brodie D (2020). Preparing for the Most critically Ill patients With COVID-19: the potential role of extracorporeal membrane oxygenation. JAMA.

[5] Gattinoni L, Coppola S, Cressoni M, Busana M, Rossi S, Chiumello D. Covid-19 Does Not Lead to a “Typical” Acute Respiratory Distress Syndrome. Am J Respir Crit Care Med. 2020 10.1164/rccm.202003-0817LE PMC7233352 32228035

[6] Varga Z, Flammer AJ, Steiger P, Haberecker M, Andermatt R, Zinkernagel AS, et al. Endothelial cell infection and endotheliitis in COVID-19. The Lancet [Internet]. 2020 [cited 2020 Apr 21] https://www.thelancet.com/journals/lancet/article/PIIS0140-6736(20)30937-5/abstract 10.1016/S0140-6736(20)30937-5 PMC7172722 32325026

[7] Gallart L, Lu Q, Puybasset L, Umamaheswara Rao GS, Coriat P, Rouby JJ (1998). Intravenous almitrine combined with inhaled nitric oxide for acute respiratory distress syndrome. The NO Almitrine Study Group.. Am J Respir Crit Care Med..

[8] Papazian L, Roch A, Bregeon F, Thirion X, Gaillat F, Saux P (1999). Inhaled nitric oxide and vasoconstrictors in acute respiratory distress syndrome. Am J Respir Crit Care Med.

[9] Wysocki M, Delclaux C, Roupie E, Langeron O, Liu N, Herman B (1994). Additive effect on gas exchange of inhaled nitric oxide and intravenous almitrine bismesylate in the adult respiratory distress syndrome. Intensive Care Med.

[10] ARDS Definition Task Force, Ranieri VM, Rubenfeld GD, Thompson BT, Ferguson ND, Caldwell E, et al. Acute respiratory distress syndrome: the Berlin Definition. JAMA. 2012. 307:2526–33. 10.1001/jama.2012.5669 22797452

[11] Papazian L, Aubron C, Brochard L, Chiche J-D, Combes A, Dreyfuss D (2019). Formal guidelines: management of acute respiratory distress syndrome. Ann Intensive Care.

[12] Koulouras V, Papathanakos G, Papathanasiou A, Nakos G (2016). Efficacy of prone position in acute respiratory distress syndrome patients: A pathophysiology-based review. World J Crit Care Med.

[13] Chen L, Del Sorbo L, Grieco DL, Junhasavasdikul D, Rittayamai N, Soliman I (2020). Potential for lung recruitment estimated by the recruitment-to-inflation ratio in acute respiratory distress syndrome a clinical trial. Am J Respir Crit Care Med..

[14] Boissier F, Katsahian S, Razazi K, Thille A, Roche-Campo F, Leon R (2013). Prevalence and prognosis of cor pulmonale during protective ventilation for acute respiratory distress syndrome. Intensive Care Med.

[15] Mekontso Dessap A, Boissier F, Leon R, Carreira S, Campo FR, Lemaire F (2010). Prevalence and prognosis of shunting across patent foramen ovale during acute respiratory distress syndrome. Crit Care Med.

[16] Vincent JL, Moreno R, Takala J, Willatts S, De Mendonca A, Bruining H, et al. The SOFA (Sepsis-related Organ Failure Assessment) score to describe organ dysfunction/failure. On behalf of the Working Group on Sepsis-Related Problems of the European Society of Intensive Care Medicine. Intensive Care Med. 1996;22:707–10. 10.1007/BF01709751 8844239

[17] Le Gall JR, Lemeshow S, Saulnier F (1993). A new Simplified Acute Physiology Score (SAPS II) based on a European/North American multicenter study. JAMA.

[18] Keyaerts E, Vijgen L, Chen L, Maes P, Hedenstierna G, Van Ranst M (2004). Inhibition of SARS-coronavirus infection in vitro by S-nitroso-N-acetylpenicillamine, a nitric oxide donor compound. Int J Infect Dis IJID Off Publ Int Soc Infect Dis.

[19] Akerström S, Mousavi-Jazi M, Klingström J, Leijon M, Lundkvist A, Mirazimi A (2005). Nitric oxide inhibits the replication cycle of severe acute respiratory syndrome coronavirus. J Virol.

[20] Chen L, Liu P, Gao H, Sun B, Chao D, Wang F (2004). Inhalation of nitric oxide in the treatment of severe acute respiratory syndrome: a rescue trial in Beijing. Clin Infect Dis Off Publ Infect Dis Soc Am.

[21] Martel J, Ko Y-F, Young JD, Ojcius DM. Could nasal nitric oxide help to mitigate the severity of COVID-19? Microbes Infect. 2020 10.1016/j.micinf.2020.05.002 PMC7200356 32387333

[22] Kobayashi J, Murata I. Nitric oxide inhalation as an interventional rescue therapy for COVID-19-induced acute respiratory distress syndrome. Ann Intensive Care [Internet]. 2020 [cited 2020 Jun 2];10. https://annalsofintensivecare.springeropen.com/articles/10.1186/s13613-020-00681-9 10.1186/s13613-020-00681-9 PMC7238394 32436029

[23] Ackermann M, Verleden SE, Kuehnel M, Haverich A, Welte T, Laenger F, et al. Pulmonary Vascular Endothelialitis, Thrombosis, and Angiogenesis in Covid-19. N Engl J Med. 2020;NEJMoa2015432. 10.1056/NEJMoa2015432 PMC7412750 32437596

[24] CRICS TRIGGERSEP Group (Clinical Research in Intensive Care and Sepsis Trial Group for Global Evaluation and Research in Sepsis), Helms J, Tacquard C, Severac F, Leonard-Lorant I, Ohana M, et al. High risk of thrombosis in patients with severe SARS-CoV-2 infection: a multicenter prospective cohort study. Intensive Care Med [Internet]. 2020 [cited 2020 May 20]; http://link.springer.com/10.1007/s00134-020-06062-x 10.1007/s00134-020-06062-x PMC7197634 32367170

[25] Lei C, Su B, Dong H, Bellavia A, Di Fenza R, Safaee Fakhr B (2020). Protocol of a randomized controlled trial testing inhaled Nitric Oxide in mechanically ventilated patients with severe acute respiratory syndrome in COVID-19 (SARS-CoV-2). Intensive Care Critical Care Med..

[26] Gattinoni L, Chiumello D, Caironi P, Busana M, Romitti F, Brazzi L, et al. COVID-19 pneumonia: different respiratory treatments for different phenotypes? Intensive Care Med. 2020 10.1007/s00134-020-06033-2 PMC7154064 32291463

[27] Cummings MJ, Baldwin MR, Abrams D, Jacobson SD, Meyer BJ, Balough EM (2020). Epidemiology, clinical course, and outcomes of critically ill adults with COVID-19 in New York City: a prospective cohort study. Lancet Lond Engl.

[28] Lang M, Som A, Mendoza DP, Flores EJ, Reid N, Carey D, et al. Hypoxaemia related to COVID-19: vascular and perfusion abnormalities on dual-energy CT. Lancet Infect Dis. 2020 [cited 2020 Jun 8]; https://linkinghub.elsevier.com/retrieve/pii/S1473309920303674 10.1016/S1473-3099(20)30367-4 PMC7252023 32359410

[29] Poissy J, Goutay J, Caplan M, Parmentier E, Duburcq T, Lassalle F, et al. Pulmonary Embolism in COVID-19 Patients: Awareness of an Increased Prevalence. Circulation. 2020; 10.1161/CIRCULATIONAHA.120.047430 32330083

[30] Ranucci M, Ballotta A, Di Dedda U, Bayshnikova E, Dei Poli M, Resta M, et al. The procoagulant pattern of patients with COVID-19 acute respiratory distress syndrome. J Thromb Haemost JTH. 2020 10.1111/jth.14854 PMC9906332 32302448

[31] Ackermann M, Verleden SE, Kuehnel M, Haverich A, Welte T, Laenger F, et al. Pulmonary Vascular Endothelialitis, Thrombosis, and Angiogenesis in Covid-19. N Engl J Med. 2020 10.1056/NEJMoa2015432 PMC7412750 32437596

[32] Menter T, Haslbauer JD, Nienhold R, Savic S, Hopfer H, Deigendesch N, et al. Post-mortem examination of COVID19 patients reveals diffuse alveolar damage with severe capillary congestion and variegated findings of lungs and other organs suggesting vascular dysfunction. Histopathology. 2020 10.1111/his.14134 PMC7496150 32364264

[33] Losser M-R, Lapoix C, Delannoy M, Champigneulle B, Payen D. Almitrine as a non-ventilatory strategy to improve intrapulmonary shunt in COVID-19 patients. Anaesth Crit Care Pain Med. 2020 10.1016/j.accpm.2020.05.013 PMC7271838 32505756

[34] Payen DM, Brun-Buisson CJ, Carli PA, Huet Y, Leviel F, Cinotti L (1985). Hemodynamic, gas exchange, and hormonal consequences of LBPP during PEEP ventilation. J Appl Physiol Bethesda Md.

[35] Barthélémy R, Blot P-L, Tiepolo A, Le Gall A, Mayeur C, Gaugain S, et al. Efficacy of Almitrine in The Treatment of Hypoxemia in Sars-Cov-2 Acute Respiratory Distress Syndrome. Chest [Internet]. 2020 [cited 2020 Jun 8]; https://linkinghub.elsevier.com/retrieve/pii/S0012369220316433 10.1016/j.chest.2020.05.573 PMC7274946 32512007

[36] Cardinale M, Esnault P, Cotte J, Cungi PJ, Goutorbe P. Effect Of Almitrine Bismesylate And Inhaled Nitric Oxide On Oxygenation In Covid-19 Acute Respiratory Distress Syndrome. Anaesth Crit Care Pain Med [Internet]. 2020 [cited 2020 Jun 8]; https://linkinghub.elsevier.com/retrieve/pii/S2352556820300990 10.1016/j.accpm.2020.05.014 PMC7271846 32505755

[37] Payen DM, Muret J (1999). Nitric oxide and almitrine: the definitive answer for hypoxemia. Curr Opin Anaesthesiol.

[38] Papazian L, Bregeon F, Gaillat F, Thirion X, Roch A, Cortes E (1999). Inhaled NO and almitrine bismesylate in patients with acute respiratory distress syndrome: effect of noradrenalin. Eur Respir J.

[39] Esnault P, Hraiech S, Bordes J, Forel J-M, Adda M, Rambaud R (2019). Evaluation of almitrine infusion during veno-venous extracorporeal membrane oxygenation for severe acute respiratory distress syndrome in adults. Anesth Analg.

[40] B’chir A, Mebazaa A, Losser MR, Romieu M, Payen D (1998). Intravenous almitrine bismesylate reversibly induces lactic acidosis and hepatic dysfunction in patients with acute lung injury. Anesthesiology.

