# Supplementary material for: Rescue therapy with inhaled nitric oxide and almitrine in COVID-19 patients with severe acute respiratory distress syndrome
Source: Ann Intensive Care. 2020 Nov 4;10:151. doi: 10.1186/s13613-020-00769-2 (PMC7641257; doi:10.1186/s13613-020-00769-2)
Supplement: Supplementary file 1 — Additional file 1: Table S1. Blood gas before and after the last proning session in ten patients with severe acute respiratory distress syndrome secondary to coronavirus disease 2019. [file 13613_2020_769_MOESM1_ESM.docx]

| **Table S2. Respiratory mechanics in supine position in ten patients with severe acute respiratory distress syndrome secondary to coronavirus disease 2019** | |
| --- | --- |
| **Variables** | **Value** |
| Tidal volume (mL/kg of PBW) | 6.5 (6.3-7.0) |
| Respiratory rate (/min) | 33 (30-35) |
| Peak inspiratory pressure (cmH_2_O) | 42 (40-43) |
| Plateau pressure (cmH_2_O) | 27 (26-29) |
| Total PEEP (cmH_2_O) | 13 (10-15) |
| Driving pressure (cmH_2_O) | 15 (12-16) |
| Crs (mL/cmH_2_O) | 28 (21-38) |
| Airway resistance (cmH_2_O.s.L^-1^) | 15 (12-17) |
| Airway opening pressure (cmH_2_O) | 2 (0-6) |
| Recruited volume (mL) | 155 (91-246) |
| Recruitment-to-Inflation ratio | 0.49 (0.27-0.77) |
| Values are expressed as median (interquartile range). PBW: predicted body weight; PEEP: positive end expiratory pressure; Crs: Respiratory system compliance; Total PEEP and plateau pressure were measured by short end-expiratory and end-inspiratory occlusions, respectively. | |
