# Supplementary figures and images for: Rescue therapy with inhaled nitric oxide and almitrine in COVID-19 patients with severe acute respiratory distress syndrome
Source: Ann Intensive Care. 2020 Nov 4;10:151. doi: 10.1186/s13613-020-00769-2 (PMC7641257; doi:10.1186/s13613-020-00769-2)

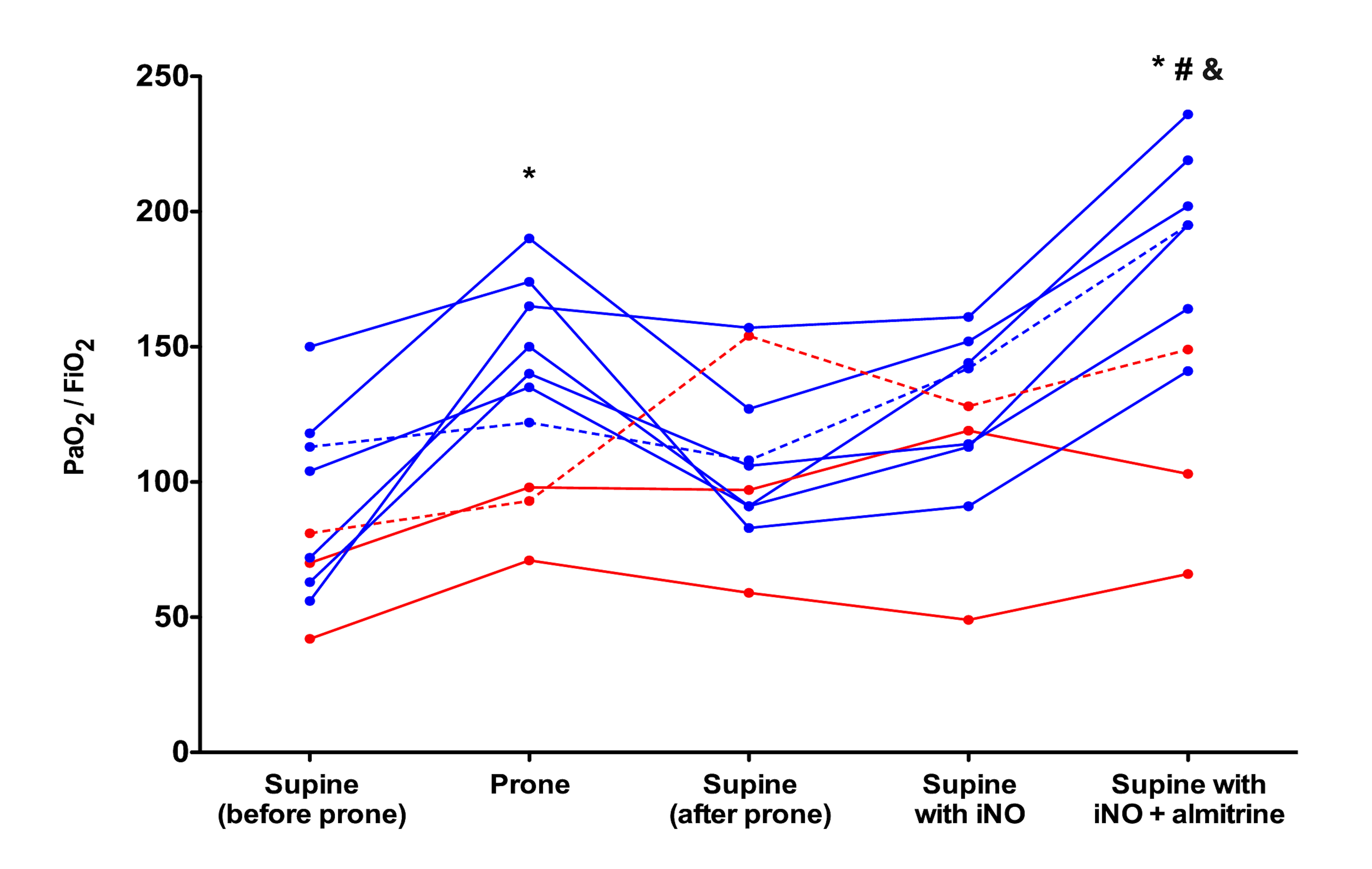

Supplement: Supplementary file 2 — Additional file 2: Figure S1. Individual values of the ratio of oxygen partial pressure to inspired oxygen fraction in arterial blood in patients with severe acute respiratory distress syndrome secondary to coronavirus disease 2019, according to position (prone or supine) and administration of inhaled nitric oxide with or without almitrine. *,# and & denote a p value <0.05 for paired Wilcoxon (with Benjamini-Hochberg correction) following Friedman test, as compared to Supine (before prone), Supine (after prone), and Supine with iNO, respectively. Red lines: “almitrine non-responders”; blue lines: “almitrine responders”; solid lines: “prone responders”; dashed lines: “prone non-responders”. [file 13613_2020_769_MOESM2_ESM.tiff]
