# Supplementary material for: Rescue therapy with inhaled nitric oxide and almitrine in COVID-19 patients with severe acute respiratory distress syndrome
Source: Ann Intensive Care. 2020 Nov 4;10:151. doi: 10.1186/s13613-020-00769-2 (PMC7641257; doi:10.1186/s13613-020-00769-2)
Supplement: Supplementary file 3 — Additional file 3: Table S2. Respiratory mechanics in supine position in ten patients with severe acute respiratory distress syndrome secondary to coronavirus disease 2019. [file 13613_2020_769_MOESM3_ESM.docx]

| **Table S3. Correlations between respiratory mechanics and oxygenation response to the combination of inhaled nitric oxide and almitrine in ten patients with severe acute respiratory distress syndrome secondary to coronavirus disease 2019** | | | |  |
| --- | --- | --- | --- | --- |
| **Variables** | **Correlation**  **coefficient** | **P value** | |  |
| Tidal volume (mL/kg of PBW) | -0.41 | 0.24 | |  |
| Respiratory rate (/min) | -0.02 | 0.96 | |  |
| Peak inspiratory pressure (cmH_2_O) | 0.06 | 0.87 | |  |
| Plateau pressure (cmH_2_O) | -0.48 | 0.16 | |  |
| Total PEEP (cmH_2_O) | -0.12 | 0.75 | | |
| Driving pressure (cmH_2_O) | -0.29 | 0.42 |  |  |
| Crs (mL/cmH_2_O) | 0.05 | 0.89 |  |  |
| Airway resistance (cmH_2_O.s.L^-1^) | 0.37 | 0.29 | |  |
| Airway opening pressure (cmH_2_O) | -0.41 | 0.23 |  |  |
| Recruited volume (mL) | 0.21 | 0.56 |  |  |
| Recruitment-to-Inflation ratio | 0.16 | 0.65 |  |  |
| PBW: predicted body weight; PEEP: positive end expiratory pressure; Crs: Respiratory system compliance; Total PEEP and plateau pressure were measured by short end-expiratory and end-inspiratory occlusions, respectively; correlation coefficients were assessed with Spearman test. | | | |  |
