# Supplementary material for: Rescue therapy with inhaled nitric oxide and almitrine in COVID-19 patients with severe acute respiratory distress syndrome
Source: Ann Intensive Care. 2020 Nov 4;10:151. doi: 10.1186/s13613-020-00769-2 (PMC7641257; doi:10.1186/s13613-020-00769-2)
Supplement: Supplementary file 4 — Additional file 4: Table S3. Correlations between respiratory mechanics and oxygenation response to the combination of inhaled nitric oxide and almitrine in ten patients with severe acute respiratory distress syndrome secondary to coronavirus disease 2019. [file 13613_2020_769_MOESM4_ESM.docx]

| **Table S1. Blood gas before and after the last proning session in ten patients with severe acute respiratory distress syndrome secondary to coronavirus disease 2019** | | | |
| --- | --- | --- | --- |
| **Variables** | **Before last**  **proning** | **End of last**  **proning** | ***p* value** |
| pH | 7.34 (7.27-7.44) | 7.35 (7.33-7.38) | 0.74 |
| PaCO_2_ (mmHg) | 46 (44-54) | 46 (42-55) | 0.92 |
| Bicarbonates (mmol/L) | 26 (24-33) | 26 (24-33) | 0.14 |
| SaO_2_ (%) | 91 (89-95) | 95 (90-97) | 0.11 |
| PaO_2_/FiO_2_ | 77 (62-114) | 137 (97-167) | <0.01 |
| Values are expressed as median (interquartile range). FiO_2_: fraction of inspired oxygen; PaO_2_: arterial oxygen tension; PaCO_2_: arterial carbon dioxide tension; SaO_2_: arterial oxygen saturation. | | | |
